# Supplementary figures and images for: Metabolome Profiling of Marrubium peregrinum L. and Marrubium friwaldskyanum Boiss Reveals Their Potential as Sources of Plant-Based Pharmaceuticals
Source: Int J Mol Sci. 2023 Dec 1;24(23):17035. doi: 10.3390/ijms242317035 (PMC10707198; doi:10.3390/ijms242317035)

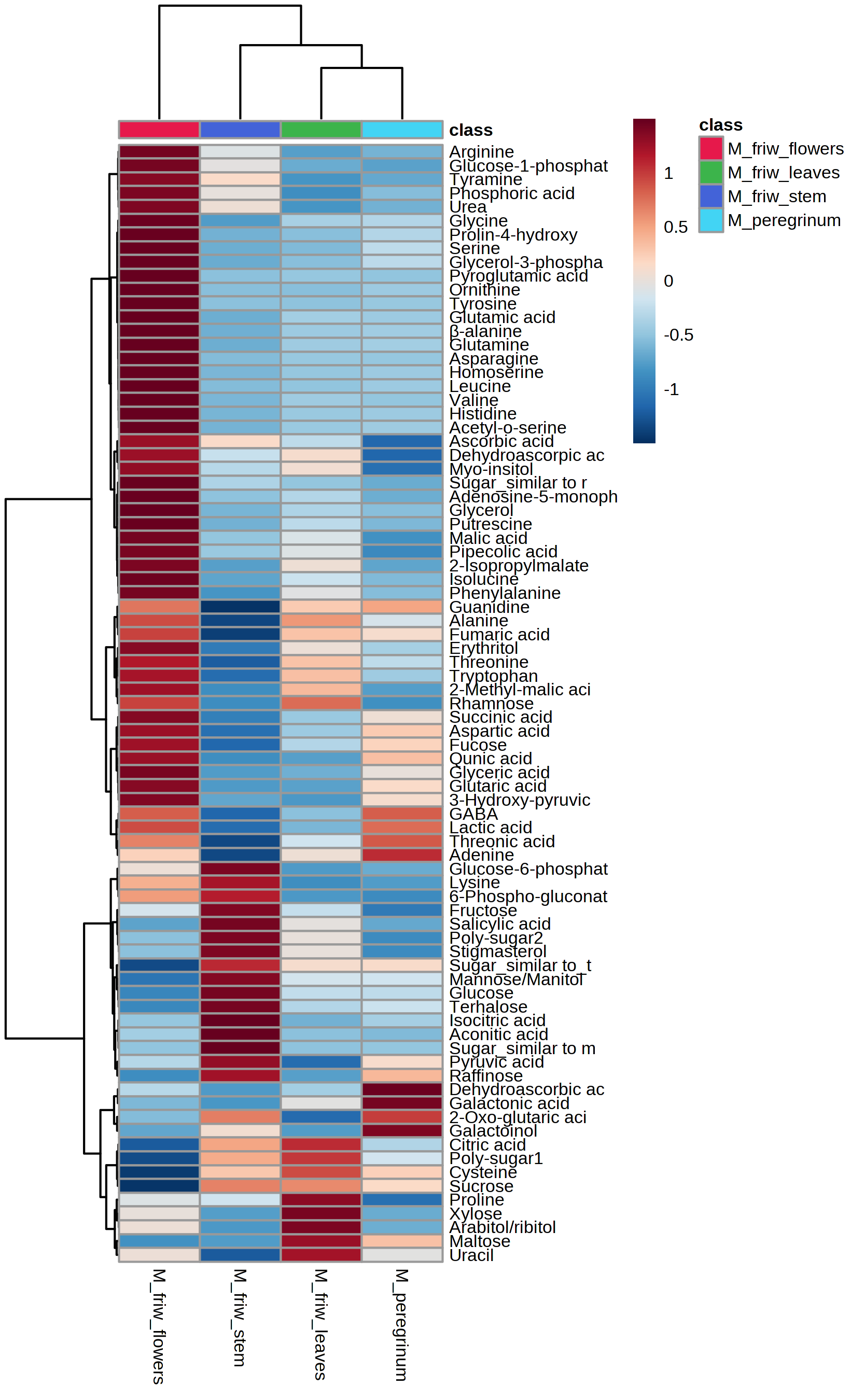

Supplement: Supplementary file 1 [file ijms-24-17035-s001.zip › Figure S1_Heatmap of the primary metabolites.png]

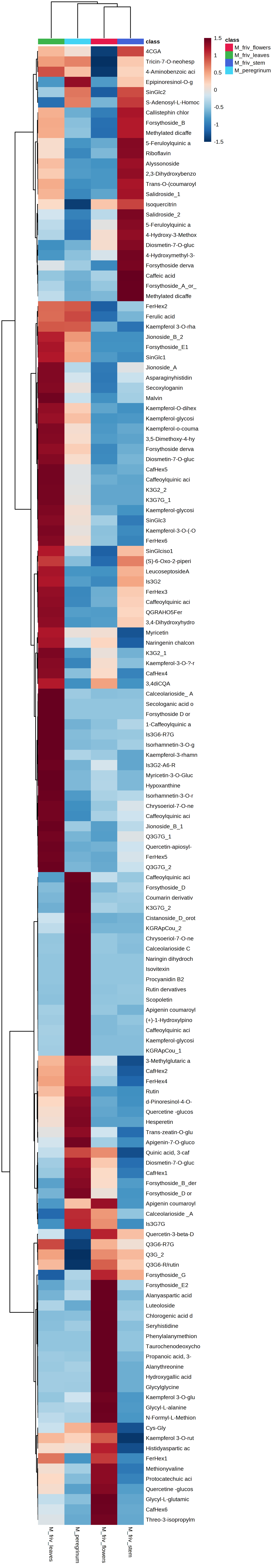

Supplement: Supplementary file 1 [file ijms-24-17035-s001.zip › Figure S2_Heatmap of the secondary metabolites.png]

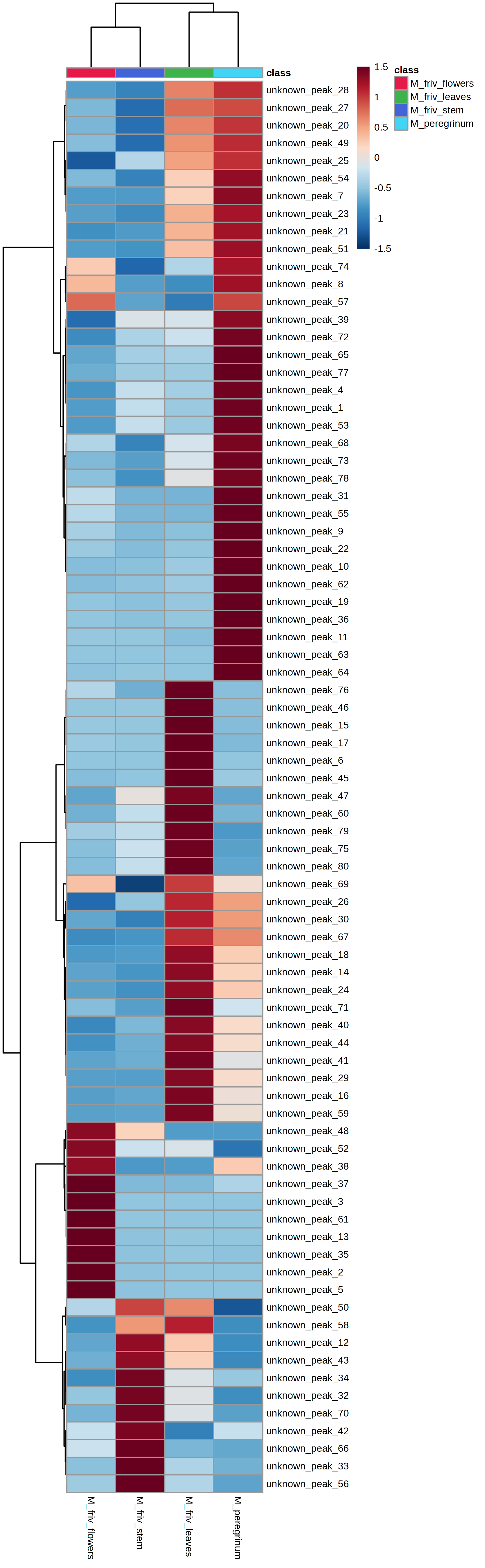

Supplement: Supplementary file 1 [file ijms-24-17035-s001.zip › Figure S3_Heatmap of the secondary unknown metabolites.png]

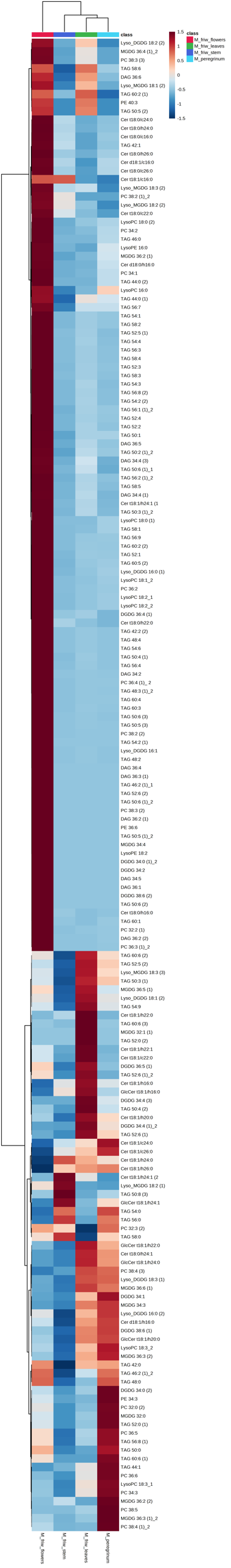

Supplement: Supplementary file 1 [file ijms-24-17035-s001.zip › Figure S4_Heatmap_Lipids.png]

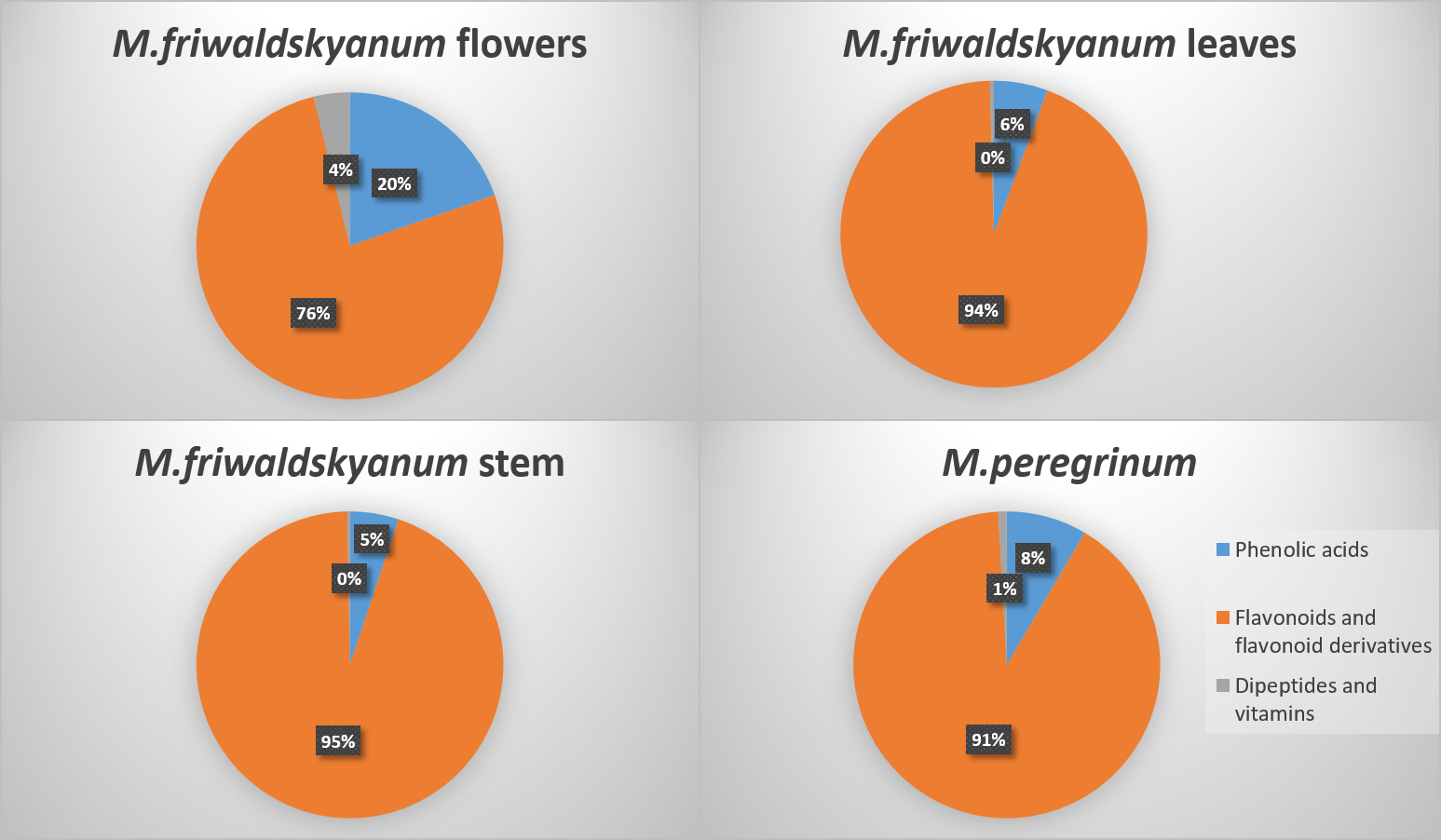

Supplement: Supplementary file 1 [file ijms-24-17035-s001.zip › Figure S5_PA_Flavonoid proportions.png]
